# Indirect Basal Metabolism Estimation in Tailoring Recombinant Human TSH Administration in Patients Affected by Differentiated Thyroid Cancer: A Hypothesis-Generating Study

**DOI:** 10.3389/fendo.2018.00037

**Published:** 2018-02-15

**Authors:** Agnese Barnabei, Lidia Strigari, Agnese Persichetti, Roberto Baldelli, Laura Rizza, Claudia Annoscia, Rosa Lauretta, Giovanni Cigliana, Maddalena Barba, Aurora De Leo, Marialuisa Appetecchia, Francesco Torino

**Affiliations:** ^1^Endocrinology Unit, “Regina Elena” National Cancer Institute of Rome, Rome, Italy; ^2^Laboratory of Medical Physics and Expert Systems, “Regina Elena” National Cancer Institute of Rome, Rome, Italy; ^3^Department of Molecular Medicine, University La Sapienza, Rome, Italy; ^4^Endocrinology Unit, Azienda Ospedaliera San Camillo – Forlanini, Rome, Italy; ^5^Laboratory Unit, “Regina Elena” National Cancer Institute of Rome, Rome, Italy; ^6^Oncology B Unit, “Regina Elena” National Cancer Institute of Rome, Rome, Italy; ^7^Medical Oncology, Department of Systems Medicine, Tor Vergata University of Rome, Rome, Italy

**Keywords:** recombinant human TSH, basal metabolism, thyroid cancer, body mass index, sex

## Abstract

**Purpose:**

Recombinant human TSH (rhTSH) is currently used in follow-up of patients affected by differentiated thyroid cancer (DTC). Age, sex, weight, body mass index, body surface area (BSA) and renal function are known factors affecting serum TSH peak levels, but the proper rhTSH dose to deliver to single patient remains elusive. In this study, the correlations of basal metabolic rates with serum TSH peak following rhTSH administration were investigated.

**Methods:**

We evaluated 221 patients affected by thyroid cancer that received a standard dose rhTSH. Blood samples were collected at pre-established time points. Data on body weight, height, and BSA were collected. The Mifflin-St Jeor and Fleisch equations were used to assess basal metabolism.

**Results:**

The median value (range) of serum TSH peaks was 142 ± 53 μU/ml. Serum TSH peaks were significantly lower in males than in females (*p* = 0.04). TSH values also increased with age. Data showed a significant decrease of TSH peak levels at day 3 from the administration of rhTSH when basal metabolic rates increased (*p* = 0.002 and *p* = 0.009, respectively). Similar findings were observed at day 5 (*p* = 0.004 and *p* = 0.04, respectively). A multivariate analysis of several factors revealed that patients’ basal metabolism (obtained using the Mifflin-St Jeor but not Fleisch equation) predicts serum TSH level peak at day 3 (*p* < 0.001). These results were used to generate a new formula based on Mifflin-StJeor equation which reveals as a promising tool in tailoring rhTSH dose.

**Conclusion:**

Basal metabolism appears an improving factor in tailoring diagnostic rhTSH dose in patients affected by DTC.

## Introduction

In patients diagnosed with early or locally advanced differentiated thyroid cancer (DTC), total thyroidectomy is currently the primary treatment and levothyroxine (LT4) at suppressive dose is required to prevent TSH-induced growth of potentially residual cancer cells (1).

In some patients, an ablative dose of radioiodine (RAI) is delivered to kill residual cancer cells, thus reducing risk of recurrence. In DTC patients, follow-up is based on serum thyroglobulin (TSH stimulated or during l-tiroxine therapy) and neck ultrasound. Diagnostic 131-radioiodine whole-body scan (131-I WBS) is not routinely used during follow-up, but may be required in specific conditions (1).

Elevated serum TSH are needed to RAI therapy and 131-I WBS execution, as well as also to evaluate stimulated thyroglobulin during follow-up. Elevated serum TSH are obtainable either through LT4 withdrawal or by the administration of recombinant human TSH (rhTSH), a hydrophilic drug, which is metabolized and eliminated by the kidney (2–5).

In DTC patients under LT4 therapy, the intramuscular administration of fixed dose rhTSH (0.9 mg/die for 2 consecutive days) increases secretion of thyroglobulin (Tg) and 131-I uptake in the case of persistent or recurrent disease (1).

Compared with LT4 withdrawal, rhTSH provides patients less side effects and better quality of life, and lower radiation exposure during 131-I WBS (6–8).

Recombinant human TSH is approved by the Food and Drug Administration and the European Medicines Agency for the ablation of residual thyroid tissue in low-risk patients who undergone total/near total thyroidectomy and in the follow-up of patients affected by DTC^1^. Serum TSH peak levels are currently assessed to measure response to rhTSH. Unfortunately, rhTSH-induced serum TSH peak levels are remarkably variable and unpredictable, being affected by several factors such as patient age, sex, height, weight, body surface area (BSA), body mass index (BMI), body composition, and renal function (4, 9, 10).

However, the heterogeneity of the above factors make challenging to define the proper rhTSH dose to prescribe to single patient (11). To this aim, a unifying tool based on known factors influencing TSH peak levels could be useful. The Mifflin-St Jeor equation and the Fleisch equation are commonly used in the assessment of basal metabolism, being the former more commonly used in obese patients (Table 1) (12, 13). In these equations, some of the most important factors influencing rhTSH-induced serum TSH peak levels (i.e., sex, age, weight, and height) are included.

**Table 1:** Formulas currently used to calculate BMI and surface area and to estimate basal metabolism rate.

	Female	Male
Body mass index (BMI) (kg/m^2^)	weight (kg)/[height (m)]^2^	weight (kg)/[height (m)]^2^
Body surface area (BSA; DuBois) (mq)	0.20247 × height (m)^0.725^ × weight (kg) ^0.425^	0.20247 × height (m)^0.725^ × weight (kg)^0.425^
**Basal metabolic rate**		
Mifflin-St Jeor equation (kcal/day)	9.99 × weight (kg) + 6.25 × height (cm) − 4.92 × age (years) − 161	9.99 × weight (kg) + 6.25 × height (cm) − 4.92 × age (years) + 5;
Fleisch equation (kcal/day)	24 × BSA × [38 − 0.073 × (age − 20)]	24 × BSA × [35 − 0.064 × (age−20)]

The present study was aimed at evaluating potential associations between basal metabolism rates and serum TSH peak levels obtained by rhTSH administration in patients affected by DTC. In particular, we tried to verify whether basal metabolism assessment could be useful in tailoring rhTSH dose in patients with DTC. In addition, we searched for an integrative formula capable to include metabolic parameters that could be useful in identifying the appropriate rhTSH dose for individual patients, in turn improving the efficacy of rhTSH stimulation (4, 9, 10, 14, 15).

## Materials and Methods

The retrospective study was based on data extracted from records of 221 consecutive Caucasian patients affected by non-metastatic DTC. All patients underwent total thyroidectomy, received a LT4 suppressive dose and were followed up with 131-I WBS. Each patient received the rhTSH standard dose (0.9 mg daily i.m. for two consecutive days) (1). Blood samples for the assessment of serum TSH peak levels were taken in overnight fasting conditions before the first rhTSH dose (day 1), and at 24, 48, and 72 h after the second rhTSH dose (days 3, 4, and 5, respectively). The local Ethical Committee approved the study. Each patient signed an informed consent, then physical examination was performed and anthropometric data were collected according to standardized methods. Body weight and height were measured in kilograms and meters, respectively. BMI was computed as weight in kilograms divided by the square of height in meters (kg/m^2^) and considered as a categorical variable according to WHO^2^. Both the Fleisch (12) and Mifflin-St Jeor (13) equations were used to assess basal metabolism. The BSA was measured according to DuBois formula (16). Serum TSH was assessed by an ultra-sensitive ECLIA assay. Serum TSH levels were all assessed in the Clinical Pathology Laboratory at the Regina Elena National Cancer Institute of Rome. As recommended, our adequate TSH peak level following rhTSH stimulation was 100 μU/ml (17).

### Statistics Analysis

Descriptive statistics were computed for all the variables of interest. Means and ranges for continuous variables while frequencies and percentage values for categorical variables were used. Existing differences between medians were evaluated using the Student’s *t*-test or one-way ANOVA test according to the number (2 or more) of groups compared. Similarly, depending upon the number and size of compared groups, we used the Pearson’s chi-squared test of independence or Fisher’s exact test (two-tailed) to assess the relationship between categorical variables. TSH values were independently plotted against gender and age. Linear regression models were used to estimate the impact of patient-related variables on serum TSH levels. To investigate the behavior of TSH over time, taking into consideration potential confounders (age, gender) and the repeated measurements per patient, we performed a multilevel regression analysis. The maximum serum TSH values achieved on day 3 and 5 were assessed in light of basal metabolism throughout regression models including sex, age, weight, and height. For the multivariate statistics, a stepwise forward multiple linear regression was performed. The results obtained by regression logistic models were used as an adjustment coefficient together with the results derived from basal metabolic assessment. Thus, an innovative formula was generated that was retrospectively tested in our study cohort. The values of doses calculated according to the new formula were compared to the delivered doses. All tests were two-sided, and differences were considered significant if *p* < 0.05. All statistics were calculated using R-Package (version3.1).

## Results

Data regarding the demographic and morphometric characteristics of the 221 patients included in the study cohort are reported in Table 2. Forty-seven patients were male and 174 female. Median age in the female and male group was 47 years (ranges 16–79 in female and 26–78 in male group, respectively; *p* = 0.80). Median BMI was 27.0 kg/m^2^ (range 19.7–36.4) and 25.8 kg/m^2^ (range 18.0–48.8) in male and female groups, respectively. Median BSA was 2.00 m^2^ (range 1.77–2.39) and 1.76 m^2^ (range 1.45–2.39) in male and female groups, respectively. The difference in BSA between males and females was statistically significant (*p* < 0.001), but not the difference in BMI (*p* = 0.882).

**Table 2:** Characteristics of patients (*N* = 221).

	Overall	Females	Males	*p* value
Sex		174	47	
Age (years), median (range)	47 (16–79)	47 (16–79)	47 (16–79)	*p* = 0.80
Body mass index (kg/m^2^), median (range)	26.1 (18–49)	25.8 (18.0–48.8)	27.0 (19.7–36.4)	*p* = 0.882
Body surface area (mq), median (range)	1.83 (1.4–2.4)	1.76 (1.45–2.4)	2.00 (1.77–2.4)	*p* < 0.001
Fleisch formula (kcal), median (range)	1,550 (1,190–2,010)	1,504 (1,191–2,100)	1,621 (1,382–1,936)	*p* < 0.001
Mifflin-St Jeor formula (kcal), median (range)	1,359 (1,044–2,251)	1,301 (1,044–1,883)	1,669 (1,371–2,251)	*p* = 0.002
Day 3 serum TSH peak levels (μU/ml), median (range)	131 (41–449)	135 (41–449)	115 (53–140)	*p* < 0.05
Day 5 serum TSH peak levels (μU/ml), median (range)	18.6 (4.5–78.0)	20 (4.5–78.0)	14 (5–45.0)	*p* < 0.05

Median basal metabolism was 1,621 kcal (range 1,382–1,936) and 1,504 kcal (range 1,191–2,100) in both male and female groups, when it was calculated according to the Fleisch equation, and 1,669 kcal (range 1,371–2,251) and 1,301 kcal (range 1,044–1,883) in male and female groups, respectively when it was according to the Mifflin-St Jeor equation. The difference in basal metabolism between male and female calculated with both the Fleisch and Mifflin-St Jeor equations was statistically significant (*p* < 0.001 and *p* = 0.002, respectively).

**Figure 1:**
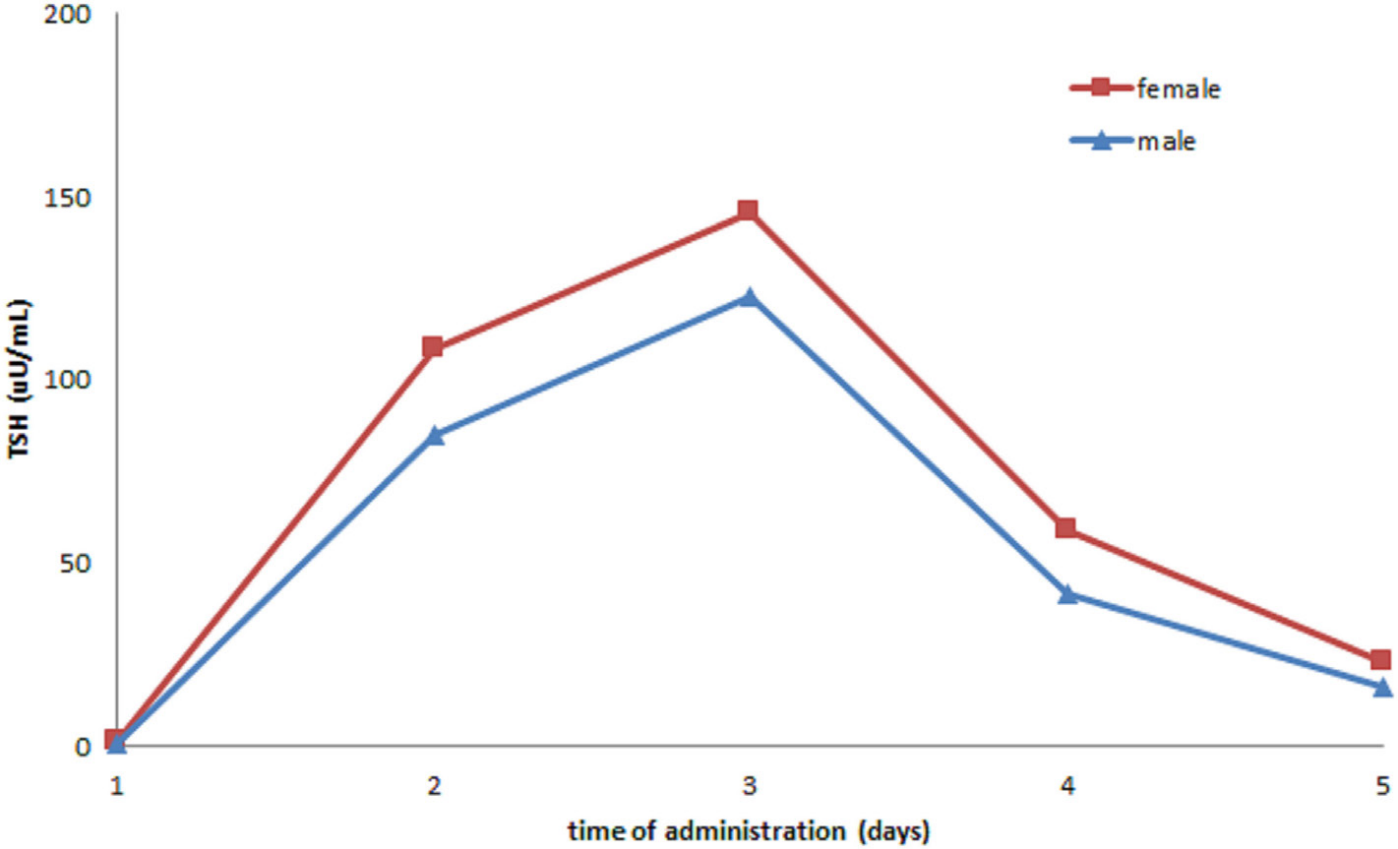
Distribution of TSH peaks by gender.

**Figure 2:**
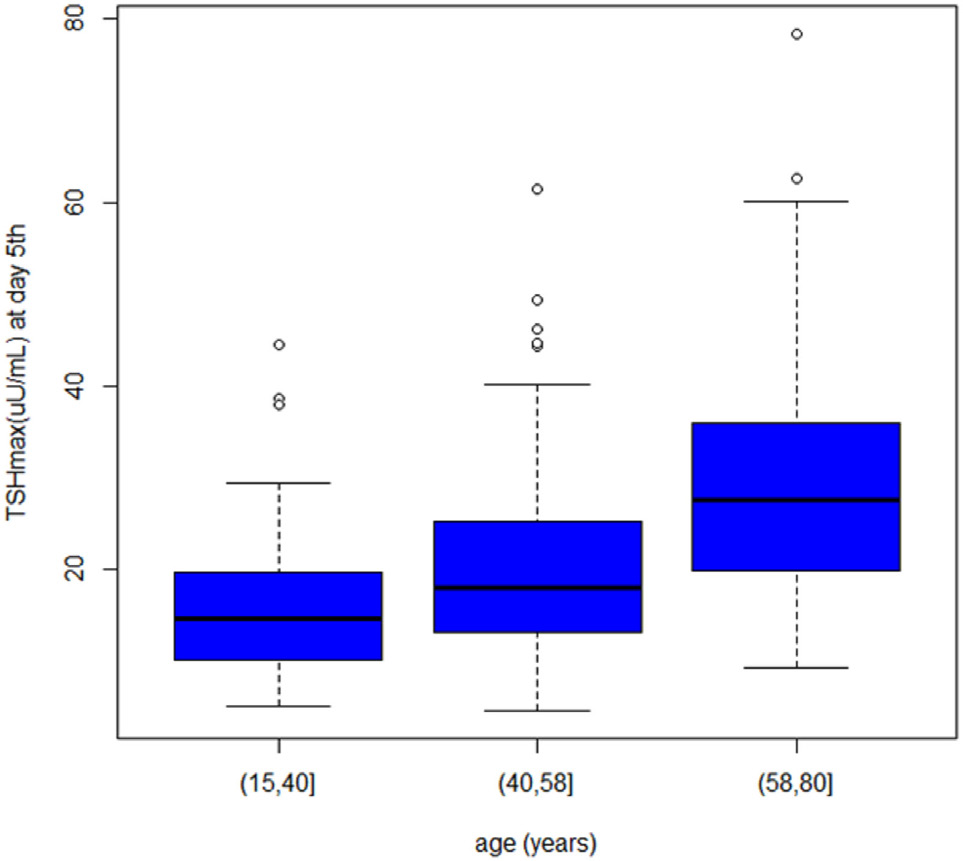
TSH peaks by age (range on day 5).

In our cohort, 269 rhTSH tests were performed. No adverse events following rhTSH administration were observed. The median value of basal serum TSH was 0.08 μU/ml (25th–75th percentile: 0.02–0.26). The median value of serum TSH peaks, assessed at day 3, was 131 μU/ml (range: 41–449). Serum TSH peaks were significantly lower in males (115 μU/ml; range 53–140) than in females (135 μU/ml; range 41–449) (*p* = 0.04) (Figure 1). Serum TSH peak levels assessed at day 5 increased significantly with age (Figure 2). Females maintained significant higher serum TSH levels than males across all the assessments. Moreover, multilevel regression analysis highlighted that TSH was higher in older (*p* = 0.007) and in the female (*p* = 0.006) patients.

When patients were stratified according to BMI, in obese patients (BMI > 30) TSH peak levels at day 3 significantly decreased, when basal metabolic rates increased (calculation according to Mifflin-St Jeor and Fleisch equations, *p* = 0.002 and *p* = 0.009, respectively) with similar results at day 5 (*p* = 0.004 and *p* = 0.04, respectively). In patients with BMI < 30, serum TSH peak levels were significantly correlated with basal metabolic rates measured with Mifflin-St Jeor equation, but not with Fleisch equation (*p* = 0.01 and *p* = 0.24, respectively).

**Figure 3:**
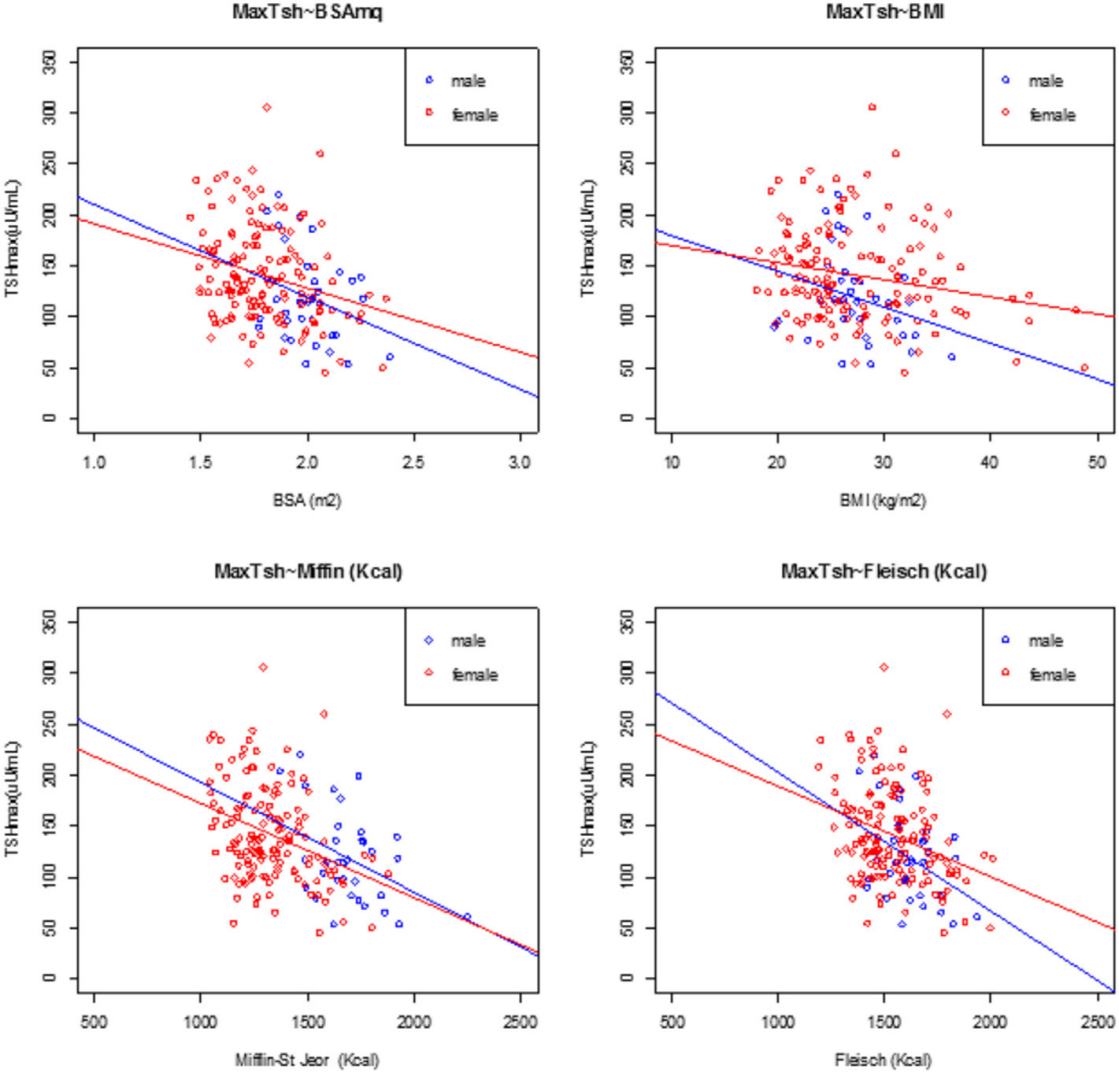
Linear regression analysis at peak TSH and body surface area (BSA), body mass index (BMI), Mifflin-St Jeor equation, and Fleisch equation at day 3, divided by male (filled circle blue) and female (open circle red).

At univariate analysis, serum TSH peak levels assessed on day 3 were inversely associated with BSA, BMI, and basal metabolism rate (BMR) (calculated according to Mifflin-St Jeor and Fleisch equations) (Figure 3), and directly related to age, with an increase of 0.8% per year. Serum TSH levels measured on the day 5 were inversely related to basal metabolism, only when calculated according to Mifflin-St Jeor equation, and directly related to age and female sex (Table 3).

**Table 3:** Univariate analysis of known factors affecting TSH peak serum levels at the third and fifth days following the administration of recombinant human TSH standard dose.

	Estimate at day 3	SE *t* value at day 3	*p*-value	Estimate at day 5	SE*t* value at day 5	*p*-value
Age	0.8797	0.2991	0.004	0.41613	0.05799	<0.001
Sex	−23.993	9.312	0.011	−5.8729	1.9222	0.002
Weight (kg)	−1.0493	0.2222	<0.001	0.007561	0.057841	0.896
Body mass index (kg/cm^2^)	−1.7659	0.6451	0.007	0.3167	0.1639	0.055
Body surface area (mq^2^)	−75.82	17.38	<0.001	−0.8913	4.5506	0.845
Fleisch formula (kcal)	−0.10778	0.02139	<0.001	−0.005868	0.005784	0.312
Mifflin-St Jeor formula (kcal)	−0.08557	0.01548	<0.001	−0.012072	0.004079	0.003

Multiple linear regression analysis (Table 4) revealed that basal metabolism results obtained using the Mifflin-St Jeor equation may predict serum TSH level peak at the day 3 (*p* < 0.001), as confirmed by Aikake Information Criteria (AIC) (18).

**Table 4:** Multivariate analysis of known factors affecting TSH peak serum levels at 3rd and 5th days following the administration of recombinant human TSH standard dose.

	Estimate at day 3	SE*t* value at day 3	*p*-value	Estimate at day 5	SE *t* value at day 5	*p*-value
Age	0.771	0.26	0.004	0.412	0.05	<0.001
Sex	−28.39	8.054	<0.001	−6.430	1.719	<0.001
Weight (kg)	−1.14	0.203	<0.001	−0.01	0.053	0.768
body mass index (kg/cm^2^)	−1.95	0.601	<0.001	0.265	0.155	0.080
Body surface area (mq)	−83.98	16.11	<0.001	−2.587	4.198	0.538
Fleisch formula (kcal)	−0.11	0.01	<0.001	−0.008	0.005	0.111
Mifflin-St. Jeor formula (kcal)	−0.092	0.0138	<0.001	−0.013	0.003	<0.001

These results may suggest that in patients with higher metabolic rates rhTSH could be more rapidly metabolized and excreted by the kidney, thus producing lower serum TSH peaks over time (Table 4).

Results obtained by regression models generated corrective factors that appeared suitable for an attempt to personalize rhTSH dose in patients affected by DTC. Using the Pearson correlation test, the logarithm of the serum TSH peak levels at the day 3 and results obtained by Mifflin-St Jeor equation appeared to be inversely related. Therefore, a new equation was generated based on the Mifflin-St Jeor equation to assess the optimal rhTSH dose to obtain the appropriate serum TSH peak level in each single patient. The new equation is reported below:

$$\text{Optimized rhTSH dose }(\text{mg})=\frac{180}{\text{exp }(5.8-0.0007\times \text{Mifflin-St Jeor equation})}$$

where the coefficient 180 in milligrams takes into account the 2-day TSH dose (0.9 mg daily for 2 days) and the elected TSH cutoff value (100 μU/ml) (17). Both 5.8 and 0.0007 are the fitted parameters describing our dataset, being 5.8 a dimensional constant, while 0.0007 is expressed in days/kcal.

According to the above equation, the calculated rhTSH dose needed to obtain the optimal TSH peak level was 1.3 mg (range 1.1–2.6). In detail, the calculated rhTSH dose was lower than that delivered in 94% of patients. In 13 (6%) patients, a higher rhTSH dose would have been needed. We estimated that 73% of patients in our cohort would have achieved the recommended rhTSH peak levels by receiving the individual dose of 1.5 mg (0.75 mg daily for 2 days), compared with 1.8 mg, the standard rhTSH dose, with 42% of patients who were estimated to have needed only 1.3 mg as rhTSH total dose (Table 5).

**Table 5:** Estimated increase or decrease in rhTSH dose to obtain appropriate TSH peak levels in the study population.

Number of patients and percentage (*n* = 221)	Delivered (standard) dose (mg)	Dose required based on the new formula
13 (6%)	1.8	Increased (more than 1.8 mg)
49 (22%)	1.8	Decreased (between 1.5 and 1.8 mg)
66 (30%)	1.8	Decreased (between 1.3 and 1.5 mg)
93 (42%)	1.8	Decreased (less than 1.3 mg)

## Discussion

In precision medicine, a still challenging issue is the tailored dosing of drugs delivered not only for therapeutic, but also for diagnostic purpose. This is the case of rhTSH, a hydrophilic drug delivered at fixed dose, having a key role in the follow-up of patients affected by DTC. As in the case of therapeutic drugs, the rhTSH delivering at fixed dose could be unsafe in a variable percentage of patients due to over or suboptimal exposure, leading in turn to vane toxic effects or inefficacy, respectively and, in both cases, causing waste of economic resource.

In patients affected by DTC, several factors have been shown to affect serum TSH peak levels following rhTSH administration, including body weight, BMI, BSA, lean body mass, and serum creatinine concentration (4, 9, 10).

Less attention has been paid to correlations between serum TSH peak levels induced by rhTSH stimulation and BMR, a parameter indicating the amount of energy (in calories) that a person needs to keep the body functioning at rest and in fasting condition.

Various tools are currently used to measure or estimate BMR, including a number of validated formulas, such as Fleish and Mifflin-St Jeor equations. Interestingly, these equations include factors that are known to influence serum TSH peak levels following rhTSH stimulation (see formula in Table 1). Based on these concepts, we hypothesized that the above equations could be used in estimating the proper dose of rhTSH to obtain optimal serum TSH peak levels in DTC patients. In our retrospective trial on a cohort 221 patients diagnosed with DTC and treated with LT4 suppressive dose, we first evaluated correlations between body weight, BMI, BSA, and BMR (measured according to Fleisch and Mifflin-St Jeor equations) and rhTSH-induced serum TSH peak levels. Then, we extrapolated a new formula exploiting a corrective coefficient and BMR to define the appropriate rhTSH dose to deliver in DTC patients. All patients received rhTSH at the standard dose of 0.9 mg for two consecutive days. Age, female sex, and BMR (according to Mufflin-St Jeor equation) significantly correlated with serum TSH peak levels. Similar results are available in the literature. In the study by Montesano et al. (15), serum TSH peak levels of 311 patients significantly correlated with gender and age and both resulted to be independent predictors of serum TSH concentrations at day 5 after rhTSH stimulation. Similarly, Over et al. in a cohort of 112 patients found a statistically significant correlation between age and serum TSH levels at day 3 and day 5 (*p* < 0.0001), compared with gender, menopausal status, weight, BMI, baseline serum TSH levels, serum creatinine, and GFR. More recently, Hautzel et al. (4) in a cohort of 286 patients found that serum TSH peak levels were negatively correlated with BSA, GFR, weight, and height and were positively related with age, female sex and serum creatinine (*p* = 0.001) at univariate analysis. However, at multivariate analysis, only BSA, renal function, sex, age, and height were all independent predictors of serum TSH peak levels on day 3. Moreover, based on these results, Hautzel et al. (4) proposed a formula aimed at predicting the TSH concentration obtainable on day 3 following rhTSH administration. All these studies suggested that multiple factors could be useful in tailoring rhTSH dose in patients affected by DTC.

In our set of patients, to evaluate potential correlations between serum TSH peak levels and BMR, we exploited two widely used formulas, i.e., Fleisch and Mifflin-St Jeor equations.

At univariate analysis, serum TSH peak levels assessed at the day 3 and 5 were found to be inversely related to BMR (calculated with Mifflin-St Jeor equation). In details, when patients were stratified according to BMI, in obese patients (BMI > 30) serum TSH peak levels at the day 3 significantly decreased when metabolic parameters increased (Mifflin-St Jeor equation *p* = 0.002 and Fleisch *p* = 0.009, respectively) with similar results at the day 5 (*p* = 0.004 and *p* = 0.04, respectively). Notably, in the group of patients with BMI > 30, only metabolic parameters measured according to Mifflin-St Jeor equation remained statistically significant (*p* = 0.004) at the day 5. In patients with BMI < 30, serum TSH levels were significantly related to BMR measured according to Mifflin-St Jeor equation, but not to Fleisch equation (*p* = 0.01 and *p* = 0.24, respectively). At the Pearson correlation test, the logarithm of the serum TSH peak levels at the day 3 and the results obtained with Mifflin-St Jeor equation were inversely related, and correlation was stronger than those obtained with Fleisch equation (Table 4). Moreover, multiple linear regression analysis revealed that BMR results obtained by Mifflin-St Jeor equation may predict serum TSH peak levels at the day 3 (*p* < 0.001), as confirmed by AIC. Therefore, hypothesized that a new formula based on BMR could be used to prescribe the optimal rhTSH dose to individual DTC patients. To test the reliability of the new formula, in our cohort, we matched the delivered rhTSH dose received by patients with the calculated dose based on the new formula. Results showed that 13 (6%) patients had received a suboptimal dose, with 208 (94%) resulting overexposed. In particular, it was estimated that approximately 2/3 (73%) of patients should have received 1.5 mg, as rhTSH cumulative dose instead of the delivered standard dose (1.8 mg), with 42% of patients who were estimated to have needed only 1.3 mg as total dose.

Resting energy expenditure (REE) indicates the amount of energy needed to maintain energy balance and is closely related (but not identical) to BMR. Many methods can assess REE, including direct and indirect calorimetry, bioelectrical impedance analysis, and a plethora of predictive equations. Indeed, about 200 multi-parametric formulas are available for the calculation of REE, but their accuracy in estimating energy requirements is not clear. However, variables such as age, weight, height, and sex are usually part of these formulas. Fleisch and Mifflin-St Jeor equation are among the most used tools to calculate basal metabolism and in estimating REE. However, the Mifflin-St Jeor equation is currently considered the most reliable indirect tool in estimating REE/BMR in both obese and non-obese individuals (19).

The rationale that prompted to evaluate basal metabolism as a factor that could potentially have a role in discriminating the appropriate rhTSH dose relays on multiple clinical aspects. The conventional treatment for DTC patients is LT4. In the cases requiring LT4 at TSH suppressive dosing, a condition of iatrogenic subclinical hyperthyroidism may raise leading to reduction in lean mass (20). Furthermore, in these patients, the lean mass may decrease further owing to long-lasting therapy. Castagna et al. (9) in a cohort of 105 patients showed that s-rhTSH peak levels were lower in men than in women and were independently associated with the lean mass (*p* < 0.0001). Morgan and Bray suggested that lean body mass is among physical parameters mostly related to hydrophilic drug distribution and rhTSH is a hydrophilic drug (21). Moreover, BSA is considered a good parameter in estimation of hydrophilic drug distribution. Similarly, hydrophilic drug dosage is affected by kidney function, particularly serum creatinine levels, which is in turn correlated with age. Indeed, age-related changes in body composition may require reduced drug dosage and water-soluble drugs are administered based on weight, height, and BSA (BSA therapeutic index).

To our knowledge, only Hautzel et al. generated a new formula functional to define the optimal rhTSH dose (4). In his work on 286 patients, multivariate regression analysis showed that gender, age, height, BSA and renal function were significant predictors of sTSH peak levels on day 3. No data are available regarding the impact of the same factors on day 5. Hautzel formula allows the calculation of individual optimal rhTSH dose based on the above parameters (4).

Our study has some limits. We retrospectively evaluated data from a clinical series, an approach that is in nature prone to bias and confounding factors. Therefore, our results only generated a research hypothesis that need to be verified in further studies. To this aim, a validation study in a larger population of patients is currently ongoing at our Institution.

Unfortunately, a number of patients in our cohort lacked serum creatinine levels. This precluded applying the Hautzel formula in our cohort of patients and any comparison between the two formulas is deferred to further studies. A further limitation might be the use of surrogate parameters for basal metabolism calculation, which was assessed throughout the use of the Mifflin-St Jeor formula. Although it is of widely use in clinical practice, more accurate assessments are obtained by calorimetry measures and DEXA.

However, our study shows some specific strengths. First, we propose the innovative use of a particularly manageable formula that might result into a significant reduction of the costs linked to the use of rhTSH. The cost of a single rhTSH test (2 consecutive daily dose of 0.9 mg i.m.) is currently of 1.129,60 euros, taking into consideration the cost per milligram of rhTSH. In our cohort, we calculate an expenditure of 249.641,60 euros. We estimated that with the optimized dosage calculated according to the new formula, we would have obtained a more accurate serum TSH peak with a considerable cost savings of about 59.748,70 euros, corresponding to 24% of the total cost. The quality of our data increases our confidence in this study results. Indeed, standardized operative procedures were applied at blood collection, handling, and storage. In addition, TSH peak levels were all centrally assessed at the institutional laboratory wherein quality controls are systematically in place. In addition, data on anthropometrics had been collected according to previously defined and pilot-tested protocols.

In conclusion, results from our study highlight the potential benefits deriving from the use of a formula including weight, height, age, and gender (Mifflin-St Jeor equation) as variables useful in assessing the optimal dose of rhTSH to deliver to DTC patients. This may have important implications in term of cost reduction and improved clinical practice. On this basis, our results encourage further research aimed at validation of our findings in future well-designed prospective studies.

## Conclusion

Our data suggest that standard dose of rhTSH may not be suitable for each individual patient. A diagnostic rhTSH dose based on patient age, sex, BMI, BSA, and basal metabolism might improve its dosing. We propose a new formula that, by including all the above parameters, reveals as a promising tool in tailoring rhTSH dose in DTC patients. This approach need to be confirmed in future prospective clinical trials.

## Ethics Statement

All subjects gave written informed consent in accordance with the Declaration of Helsinki. The study was approved by the “Ethics Committee of Bietti Foundation - Section I.F.O. (Regina Elena National Cancer Institute and San Gallicano Dermatological Institute)”.

## Author Contributions

MA: coordination of the working group. AB, SL, and AP: study conception and design. LS, AB, and FT: analysis and interpretation of data. AP, RB, LR, CA, RL, and ADL: data collection. GC: sample data collection. FT and MB: drafting of manuscript.

## Conflict of Interest Statement

The authors declare that the research was conducted in the absence of any commercial or financial relationships that could be construed as a potential conflict of interest.

## Footnotes

^1^Available from: http://www.ema.europa.eu/docs/en_GB/document_library/EPAR_-_Product_Information/human/000220/WC500038750.pdf

^2^Available from: http://apps.who.int/bmi/index.jsp?introPage=intro_3.html
